# New Truths in Ophthalmology

**Published:** 1894-01

**Authors:** 


New Truths in Ophthalmology, as Developed by G. C. Savage, M. D., Professor of Opthalmology in the Medical Department of the University of Nashville and Vanderbilt University. Thirty-two illustrations. Published by the author. Printed at the Publishing House of the M. E. Church, South, Nashville, Tenn., 1893.

This little volume, publishhd by Dr. Savage, contains, in a great part, the papers before published by the same author in other Ophtalmological journals, subjects upon which the author has bestowed much original thought. The work is a most valuable appendix for the library of every ophthalmologist, for the subjects treated are just the ones he must contend with in every day office work. I feel that we all should thank Dr. Savage for this little work and for the clearness and terseness with which he handles the subjects. C. D. R.

